# Acupuncture with or without moxibustion for primary dysmenorrhea: A protocol for a systematic review and meta-analysis

**DOI:** 10.1097/MD.0000000000022395

**Published:** 2020-09-18

**Authors:** Xingchen Zhou, Jun Xiong, Zhenhai Chi, Fanghui Hua, Lunbin Lu, Jun Chen, Genhua Tang, Siyuan Zhu, Zhiying Zhong, Han Guo

**Affiliations:** a The Affiliated Hospital of Jiangxi University of Traditional Chinese Medicine; b Jiangxi University of Traditional Chinese Medicine, Nanchang, China.

**Keywords:** primary dysmenorrhea, acupuncture, moxibustion, systematic review, meta-analysis, protocol

## Abstract

**Background:**

Primary dysmenorrhea (PD) occurs during menstrual cramps, and there is currently no pathological evidence. This disease severely affects the daily lives of young women. Acupuncture (ACU) and moxibustion are an excellent way to relieve the pain of patients with PD. And it has been widely utilizing. However, the effectiveness and safety of ACU and moxibustion in treating patients with PD are not confirmed by a high-quality meta-analysis. This work aims to evaluate ACU's efficacy and safety with or without moxibustion in the management of PD.

**Methods:**

We will make a comprehensive retrieval in 9 databases as following: Embase; Cochrane Library; PubMed; Chinese databases SinoMed (previously called the Chinese Biomedical Database); Chinese National Knowledge Infrastructure; Chinese Scientific Journals Database; Wanfang Data. The time is limited from the construction of the library to August 2020. No restrictions about language and status. Our 2 authors will perform the selection of studies, the extraction of data, and the quality assessment with the risk of bias tool independently. We will use NoteExpressV3.2.0 and Excel2010 software to extract data. The content will be saved in electronic form. We will use the bias risk tool provided by the Cochrane Collaboration to evaluate the quality of the literature using RevMan 5.4 software. The primary outcome is the pain degree evaluation, including visual analog scale, numerical rating scale, Cox retrospective symptom scale, or any other scale used to evaluate the level of pain.

Furthermore, the response rate involved an overall reduction in symptoms. The adverse effects and quality of life will be assessed as secondary outcomes. The risk ratio for dichotomous data and mean differences with a 95% confidence interval for continuous data will be adopted to express the effect and safety of ACU with or without moxibustion for PD.

**Results:**

The results of our study expect to provide high-quality, evidence-based recommendations on further treatment for clinicians.

**Trial registration number:**

INPLASY202080006.

**Conclusion:**

This study will provide scientific evidence of PD Systematic review.

## 1 Introduction

Primary dysmenorrhea (PD) is a typical gynecological disease characterized by abdominal pain before or during menstruation.^[1]^ Patients with PD have associated nausea, vomiting, diarrhea, and even syncope.^[2]^ According to reports, the prevalence of PD ranges from 16% to 91% of women of childbearing age and 2% to 29% of severe pain.^[3]^ PD is known to have a substantial negative impact on the woman's quality of life,^[4]^ school or work attendance, and social or physical activity.^[5]^ Therefore, it is necessary to research the loss of labor caused by PD and improve the quality of life of patients.

The patient suffers great pain but it is often regarded as a normal phenomenon during menstruation and is not taken seriously by everyone. The pathophysiology of PD is based on the increased secretion of prostaglandin, which induces uterine contraction and reduces uterine blood flow.^[6]^ It is well known that uterine contractions and reduced uterine blood flow can cause pain in women with PD.^[6]^

Western medicine commonly used in the treatment of PD includes nonsteroidal anti-inflammatory drugs (NSAIDs), oral contraceptives, transcutaneous electrical nerve stimulation, and tropical heat.^[1,7,8]^ However, the side effects and drug resistance of these therapies are also troublesome. According to evidence-based medicine, NSAIDs are considered the first-line treatment for PD due to their inhibitory action on prostaglandin formation.^[9]^ But it has been reported to have various side effects on the liver, kidneys, and digestive tract.^[10]^ Therefore, the application of nonpharmaceutical intervention therapy has been paid more and more attention by researchers to relieve pain and improve the quality of life, such as ACU and moxibustion, which is generally considered safe and efficacious by the populace.

According to the theory of traditional Chinese medicine, PD's occurrence is related to the regulation of the 2 meridians of Chong and Ren channels and the regulation of uterine qi and blood. The primary manifestation is the sharp change of Qi and blood in the uterus, causing pain. Therefore, “pain caused by impediment” and “pain caused by lack of nutrition” can best represent PD's etiology and pathogenesis.^[11]^ The modern research and clinical experience of TCM doctors also show that stagnation of liver Qi, physical weakness, and invasion of wind, cold, and dampness are the root causes of dysmenorrhea.^[12,13]^

ACU and moxibustion are a characteristic therapy of TCM. The latest disease spectrum shows that PD is the dominant disease of ACU and moxibustion.^[14]^ This is mainly due to its analgesic mechanism. A study found that painful stimulation can significantly increase the concentration of free Ca^2^^+^ in the central gray matter of the rat brain, whereas ACU and moxibustion can significantly reduce its concentration.^[15]^ This reveals the analgesic mechanism of acupuncture theory.

Many randomized controlled trials (RCTs) have confirmed the efficacy of ACU or moxibustion in PD treatment.^[16,17]^ Many meta-analyses also show that ACU or moxibustion treatment has specific benefits for PD patients.^[18–20]^ However, to date, no rigorously designed meta-analysis has been conducted to evaluate a systematic review of acupuncture with or without moxibustion for PD. Therefore, the purpose of this review is to explore the clinical efficacy and sufficient evidence of ACU with or without moxibustion in the treatment of PD based on the existing systematic review (SR) methodology and report quality and to provide clinical medicine with the credibility and reliability of current evidence Information on future research directions.

## 2 Methods

### 2.1 Study registration

This protocol report is structured according to the Preferred Reporting Items for Systematic Reviews and Meta-analysis Protocols (PRISMA-P) statement.^[21]^ It is registered on the International Prospective Register of Systematic Reviews (registration number INPLASY202080006; https://inplasy.com/inplasy-2020-8-0006/).

### 2.2 Inclusion criteria

#### 2.2.1 Type of study

All the RCTs which is stated the “randomization” phrase will be included, regardless of allocation concealment or use of blinding, and published or unpublished RCTss without language restriction.

#### 2.2.2 Types of participants

This study will employ the diagnostic standards of the Clinical Guideline of PD by the Society of Obstetricians and Gynecol ogists of Canada.^[22]^ Such as endometriosis, uterine fifibroids, and adenomyosis, was not taken in consideration in this study.

#### 2.2.3 Types of Interventions

We will include trials that apply acupuncture with or without moxibustion, such as moxibustion, catgut embedding, electroacupuncture, transcutaneous electrical acupoint stimulation, auricular acupuncture, scalp acupuncture, warm needling, manual acupuncture, acupoint injection, regardless of needling techniques, and stimulation method.

#### 2.2.4 Types of control groups

The control group is treated with sham-acupuncture, placebo, and pharmacotherapy, recommended in international or domestic authorized clinical guidelines, or no treatment. When studies combine ACU treatments with other active therapy, both the experimental and the control groups are required to use the same active therapy.

#### 2.2.5 Outcomes

##### 2.2.5.1 Primary outcome measures

Visual analog scale is a 100-mm long horizontal line with 2 endpoints marked as “no pain” and “worst pain imaginable,” and it is the most frequently used instrument to measure the pain scale ofdysmenorrhea.^[23]^

##### 2.2.5.2 Secondary outcomes

Secondary outcomes will contain:

1. Quality of life measured with valid questionnaires.
2. Adverse events.

### 2.3 Exclusion criteria

The exclusion criteria contain the following items:

1. Patients with organic disease conditions or pregnancy.
2. The intervention combined with any complementary therapy will be excluded, for example, Chinese herb decoction and other complementary therapy.
3. Non-RCTs reviews, animal experiments, case reports, expert experience, and conference articles.
4. Incomplete data or information.
5. Repeatedly checked or published literature.

### 2.4 Search strategy

We plan to search the following databases: Embase; Cochrane Library; PubMed; Chinese databases SinoMed (previously called the Chinese Biomedical Database); Chinese National Knowledge Infrastructure; VIP Database for Chinese Technical Periodicals; Wanfang Data. All of them will be searched from inception to August 2020. The retrieval mode used will be a combination of free words and medical subject headings terms, including “dysmenorrhea,” “primary dysmenorrhea,” “menstrual pain,” “painful menstruation,” “period pain,” “painful period” “cramps,” “menstrual disorder,” “pelvic pain” “oligomenorrhea,” “meralgia,” “moxibustion,” “Thunder-fire miraculous moxa roll,” “Thunder fire moxibustion,” “taiyi miraculous moxa roll,” “suspended moxibustion,” “mild moxibustion,” “needle warming moxibustion,” “acupuncture,” “acupuncture therapy,” “electroacupuncture,” “auriculotherapy,” “acupoint,” “acupoint injection,” “acupoint catgut embedding,” “moxa,” “needle,” “warm needle,” “temperature needle” “randomized controlled trial,” “random allocation, ”allocation, random,“ ”RCT randomized controlled,“ ”randomized, controlled,“ ”clinical trial." The search strategy takes PubMed as an example, as shown in Table 1.

**Table 1:**
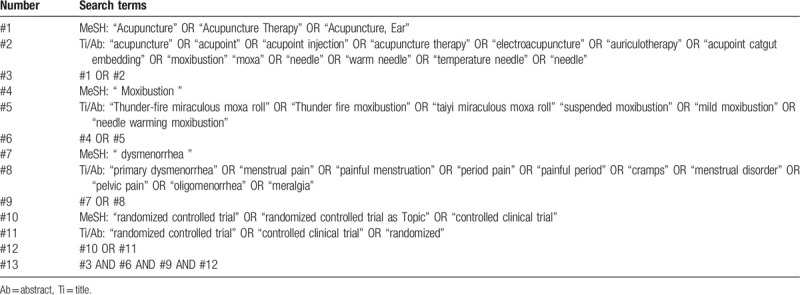
Search Strategy (PubMed).

### 2.5 Data collection and analysis

#### 2.5.1 Selection of studies

**Figure 1:**
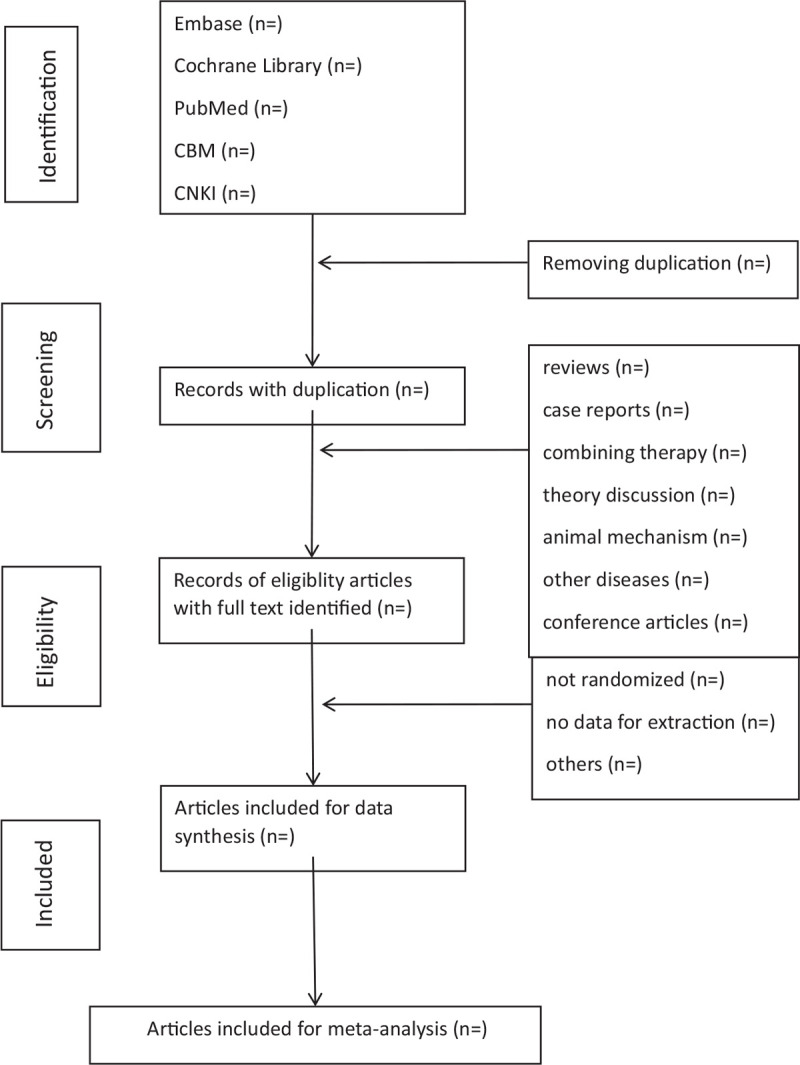
Flowchart of literature selection.

The selection, which includes literature screening, data extraction, and examination, will be conducted by 2 reviewers (ZHC and JX). If there is any disagreement, a third reviewer (LBL) will participate in the consultation to reach a consensus. The titles and abstracts of retrieved studies should be read first to rule out unrelated or repeated studies. Then, the remaining articles will be reviewed in full text by the reviewer according to the inclusion criteria, and the researches that meet the criteria will be selected at last. We will use the PRISMA-P flowchart to represent the complete process shown in the PRISMA-P flow chart (Fig. 1).

#### 2.5.2 Data extraction and management

Two independent reviewers (ZHC and XCZ) shall design a standardized data extraction form, which includes identification information (author, working location, publication date, publication source, among others), characteristic of the trial (number of groups and participants, randomized method, blinding, among others), participants (age, sex, original disease, country, diagnosis, duration, among others), interventions of both the observation group and control group (type of acupuncture, frequency, session, duration), outcomes (primary outcome and secondary outcome, time of measurement, duration of follow-up, among others), adverse effects, duration of follow-up, type and source of financial support. In case of any discrepancies between two reviewers, we will discuss and reach a consensus and seek arbitration from a third reviewer (JX).

#### 2.5.3 Dealing with missing data

If there are ambiguous or unreported data, we will contact the corresponding authors for the missing data to get specific information by telephone or email. If the missing information still cannot be obtained that missing information, we will exclude it from the analysis.

### 2.6 Risk of bias assessment

The assessment will be conducted by two reviewers (GUT and SYZ) with the risk-of-bias assessment method from Cochrane Reviewer's Handbook 5.0.24.^[24]^ The main contents comprise 7 items: random sequence generation, allocation concealment, blinding of participants and personnel, blinding of outcome assessment, incomplete outcome data, selective reporting, and bias. The studies will be evaluated as being of “low risk of bias,” “high risk of bias,” or “unclear risk of bias.” Inconsistency will be consulted with the third review author (ZYZ).

### 2.7 Statistical analysis

We will conduct statistical analyses by RevMan 5.4 software. Continuous outcomes will be pooled with standardized mean differences (SMDs) with 95% confidence interval (95% CI), and dichotomous outcomes will be analyzed by calculating odds ratios (ORs) with 95% CI. The *I*^2^ statistic will be used to assess levels of the heterogeneity of each pairwise comparison. If *I*^2^ <50%, the fixed-effect model will be used, or else a random-effect model will be used. When the results are substantial heterogeneity or considerable heterogeneity, sensitivity analysis or meta-regression and subgroup analysis will be made to explore possible sources. When there is no explanation for statistical heterogeneity, a random-effects model will be used with a test level of a = 0.05.

### 2.8 Sensitivity analysis and subgroup analysis

Sensitivity analysis will be conducted to test the robustness of critical decisions made during the review process. The central decision nodes include method quality, sample size, and the impact of missing data.

### 2.9 Subgroup analysis

If there are plenty of subgroup studies, subgroup analysis will be analyzed to determine the heterogeneity. The subgroup analysis criteria are as follows:

1. treatment time or the dose of ACU with or without moxibustion
2. duration or severity of PD
3. syndrome differentiation
4. formulations

### 2.10 Publication bias

Publication bias will be evaluated using an Egger regression test, which will help avoid observation bias and produce a funnel plot indicating a digitally based modeling result.

## 3 Discussion

PD is a common disease in women. In severe cases, it can affect patients’ life and work, bringing economic and social burdens. ACU and moxibustion as an effective technique of TCM, have been accepted for PD in China. However, due to the lack of direct comparison between different ACU and moxibustion methods, clinicians cannot choose the best one. As a result, clinicians tend to combine several physical therapies from their experience to determine which are most suitable for patients, which increases the burden of time and capital input, and results in inefficient utilization of medical resources. Therefore, this study is meant to summarize the evidence of ACU and moxibustion therapy for PD from various RCTs.

The study also has some defects as follows: low quality of original researches, the possible occurrence of false positive or false negative results, various duration of disease, different dosage, and frequency of intervention, language restriction, and so on. All of these will lead to some bias and influence the results of evaluation results, which may ultimately affect this study's reliability.

## Author contributions

All authors have read and approved the publication of the protocol.

**Conceptualization:** Xingchen Zhou, Jun Xiong.

**Data curation:** Zhenhai Chi, Fanghui Hua, Lunbin Lu, Jun Chen, Genhua Tang, Siyuan Zhu, Zhiying Zhong, Han Guo, Xingchen Zhou.

**Formal analysis:** Zhenhai Chi, Fanghui Hua.

**Investigation:** Jun Xiong, Zhenhai Chi.

**Methodology:** Lunbin Lu, Jun Chen, Genhua Tang.

**Software:** Zhenhai Chi, Siyuan Zhu.

**Supervision:** Jun Xiong, Zhenhai Chi, Zhiying Zhong.

**Writing – original draft:** Xingchen Zhou, Lunbin Lu, Han Guo.

**Writing – review & editing:** Jun Xiong, Zhenhai Chi.

## Abbreviations

Abbreviations: ACU = acupuncture, CI = confidence interval, COX = Cox retrospective symptom scale, NRS = numerical rating scale, NSAIDs = nonsteroidal anti-inflammatory drugs, OCs = oral contraceptives, PD = primary dysmenorrhea, PRISMA-P = Preferred Reporting Items for Systematic Reviews and Meta-analysis Protocols, PRO scale = patient-reported outcome scale, Qol = quality of life, RCTs = randomized controlled trials, SMDs = standardized mean differences, TCM = Traditional Chinese Medicine, TENS = transcutaneous electrical nerve stimulation, VAS = visual analog scale.
